# Renunciation of Quackery by an Apothecary

**Published:** 1848-02

**Authors:** 


*Renunciation of Quackery by an Apothecary.*—Under this caption the editor of the St. Louis Medical and Surgical Journal introduces a letter from an apothecary of St. Louis, from which we quote the following paragraph:—

"Noticing an article in the July and August Nos. of the St. Louis Medical and Surgical Journal, headed 'apothecaries dealing in quack medicines,' and, being convinced of the justice of your remarks, I have come to the determination no longer to deal in secret or patent medicines, but to confine myself to the dispensing of physician's prescriptions."

We are glad to see a precedent, which, we trust, will lead to a like renunciation by others. As a question of casuistry the business of vending secret nostrums will not bear discussion. The reflecting, candid, conscientous mind has only to admit the question fairly as a subject of deliberation, to be convinced of its moral merits. The principle involved is not that which may be urged with reference to the sale of ardent spirits, viz., that they are *often* applied to vicious indulgencies. The objections to patented nostrums are not directed against their abuse, but against their use. The system is based upon deception and fraud. The inebriate, when he receives the intoxicating cup, does so voluntarily, with a view to the debasing effects of the draught. He who offers the poisoned chalice may imagine that he is screened from blame by the fact that the responsibility of the crime rests with the recipient. We will not discuss the merits of this plea. We adduce it to show that the vender of quack remedies has not the benefit of whatever validity or force it may be thought to possess. The purchaser, in the latter case, is allured by his anxiety to recover health. He is persuaded by affidavits, certificates, and recommendations, that he will be benefitted by subjecting his system to the operation of the secret remedy. It is fully understood among apothecaries how these testimonials are obtained—they know how much value is to be attached to them—they cannot shut their eyes to the injury to life and health occasioned by the enormous consumption of patent medicines—in short they cannot but understand the true nature of the *patent medicine system*—without farther argument, then, we would commend the subject to their attention as a matter of conscience.

